# Quantification of lumbar vertebral fat deposition: Correlation with menopausal status, non-alcoholic fatty liver disease and subcutaneous adipose tissue

**DOI:** 10.3389/fendo.2022.1099919

**Published:** 2023-01-12

**Authors:** Chu-Shan Zheng, Hui-Quan Wen, Wu-Sheng Lin, Xiao-Wen Luo, Li-Shan Shen, Xiang Zhou, Feng-Yun Zou, Qing-Ling Li, Hui-Jun Hu, Ruo-Mi Guo

**Affiliations:** ^1^ Department of Radiology, Third Affiliated Hospital of Sun Yat-sen University, Guangzhou, China; ^2^ Department of Radiology, Sun Yat-Sen Memorial Hospital, Sun Yat-sen University, Guangzhou, Guangdong, China; ^3^ Department of VIP Medical Center, Third Affiliated Hospital of Sun Yat-sen University, Guangzhou, Guangdong, China

**Keywords:** magnetic resonance imaging, subcutaneous fat, severe fatty liver, postmenopausal women, fat fraction

## Abstract

**Purpose:**

To assess abdominal fat deposition and lumbar vertebra with iterative decomposition of water and fat with echo asymmetry and least-squares estimation (IDEAL-IQ) and investigate their correlation with menopausal status.

**Materials and Methods:**

Two hundred forty women who underwent routine abdominal MRI and IDEAL-IQ between January 2016 and April 2021 were divided into two cohorts (first cohort: 120 pre- or postmenopausal women with severe fatty livers or without fatty livers; second cohort: 120 pre- or postmenopausal women who were obese or normal weight). The fat fraction (FF) values of the liver (FF_liver_) and lumbar vertebra (FF_lumbar_) in the first group and the FF values of subcutaneous adipose tissue (SAT) (FF_SAT_) and FF_lumbar_ in the second group were measured and compared using IDEAL-IQ.

**Results:**

Two hundred forty women were evaluated. FF_lumbar_ was significantly higher in both pre- and postmenopausal women with severe fatty liver than in patients without fatty livers (premenopausal women: *p* < 0.001, postmenopausal women: *p* < 0.001). No significant difference in the FF_lumbar_ was observed between obese patients and normal-weight patients among pre- and postmenopausal women (premenopausal women: *p* = 0.113, postmenopausal women: *p* = 0.092). Significantly greater lumbar fat deposition was observed in postmenopausal women than in premenopausal women with or without fatty liver and obesity (*p* < 0.001 for each group). A high correlation was detected between FF_liver_ and FF_lumbar_ in women with severe fatty liver (premenopausal women: r=0.76, p<0.01; postmenopausal women: r=0.82, p<0.01).

**Conclusion:**

Fat deposition in the vertebral marrow was significantly associated with liver fat deposition in postmenopausal women.

## Introduction

Osteoporosis is characterized by reduced bone density, increased bone fragility, and susceptibility to fracture. The reduced bone density occurring in individuals with osteoporosis is associated with an increase in vertebral bone marrow fat deposition (1, 2). Age is associated with changes in the musculoskeletal system. Substantial decreases in skeletal muscle function and bone density occur with aging (3, 4).The yellow bone marrow gradually replaces the red bone marrow in the vertebral body with increasing age (3, 5). Estrogen is another important factor affects bone composition. A reduction in estrogen levels promotes bone loss and the development of osteoporosis (6). Thus, osteoporosis is common in elderly individuals, especially in postmenopausal women (7). Furthermore, accumulating evidence has shown that postmenopausal women have increased abdominal adiposity, including subcutaneous adipose tissue (SAT) and visceral adipose tissue (VAT), compared to premenopausal women (8, 9). According to recent studies, abdominal adiposity is associated with osteopenia and osteoporosis (10, 11). Proinflammatory cytokines, such as tumor necrosis factor-α and interleukin-6, secreted by VAT are known to promote bone metabolism and resorption (12). Non-alcoholic fatty liver disease (NAFLD) is a multiple-system disease that is strongly associated with abdominal obesity (13), and VAT may cause NAFLD (14). Preliminary evidence also suggests that NAFLD may be associated with a decrease in bone mineral density (15). However, the relationship between vertebral bone marrow fat and abdominal adiposity, especially SAT and liver fat, remains unknown. And the possible contribution of abdominal adiposity to osteoporosis in postmenopausal women has not been well characterized.

MRI methods are the most accurate noninvasive techniques for quantifying body fat and bone marrow fat. MR spectroscopy is the most commonly used method to examine quantitative fat measurements (16). However, it has drawbacks, such as a long scan time, small imaging range, and substantial postprocessing, which may be impractical in some clinical settings (17, 18). Iterative decomposition of water and fat with echo asymmetry and least-squares estimation (IDEAL-IQ) imaging is a new method that steadily separates fat and water using three asymmetric echo times and the three-point Dixon method. In IDEAL-IQ, an iterative least-squares decomposition algorithm is employed to solve for a fat fraction map, a water fraction map, and an R2* map simultaneously (17, 18). By incorporating an R2* map into the algorithm, IDEAL-IQ accounts for T2* effects/field inhomogeneity and yields a proton density fat fraction that is not confounded by iron overload (19–21). IDEAL-IQ has been reported to accurately quantify hepatic fat deposition with good correlations observed between hepatic MR spectroscopy and liver biopsy (22–24). It was also used to measure the fat content in other organs and tissues, such as the pancreas, kidney, and bone marrow, in individuals with NAFLD (25, 26). IDEAL-IQ has been considered a valuable tool for providing information on fat content in clinical settings.

Therefore, using IDEAL-IQ as the noninvasive imaging methodology, we aimed to investigate the correlation between SAT, NAFLD, and vertebral bone marrow fat in a population of middle-aged females and investigate the correlations between fat deposits and menopausal status.

## Materials and methods

### Subjects

A total of 2360 middle-aged females (age range, 45 - 55 years old) who had received an abdominal MRI from January 2016 to April 2021 presented for a medical examination. The final data were acquired retrospectively from 240 middle-aged female subjects (age range, 45 – 55 years old; mean age, 48.50 ± 3.54 years old). Our institutional research ethics committee approved this study (02-005-01), and written informed consent was obtained from all study participants.

**Figure 1:**
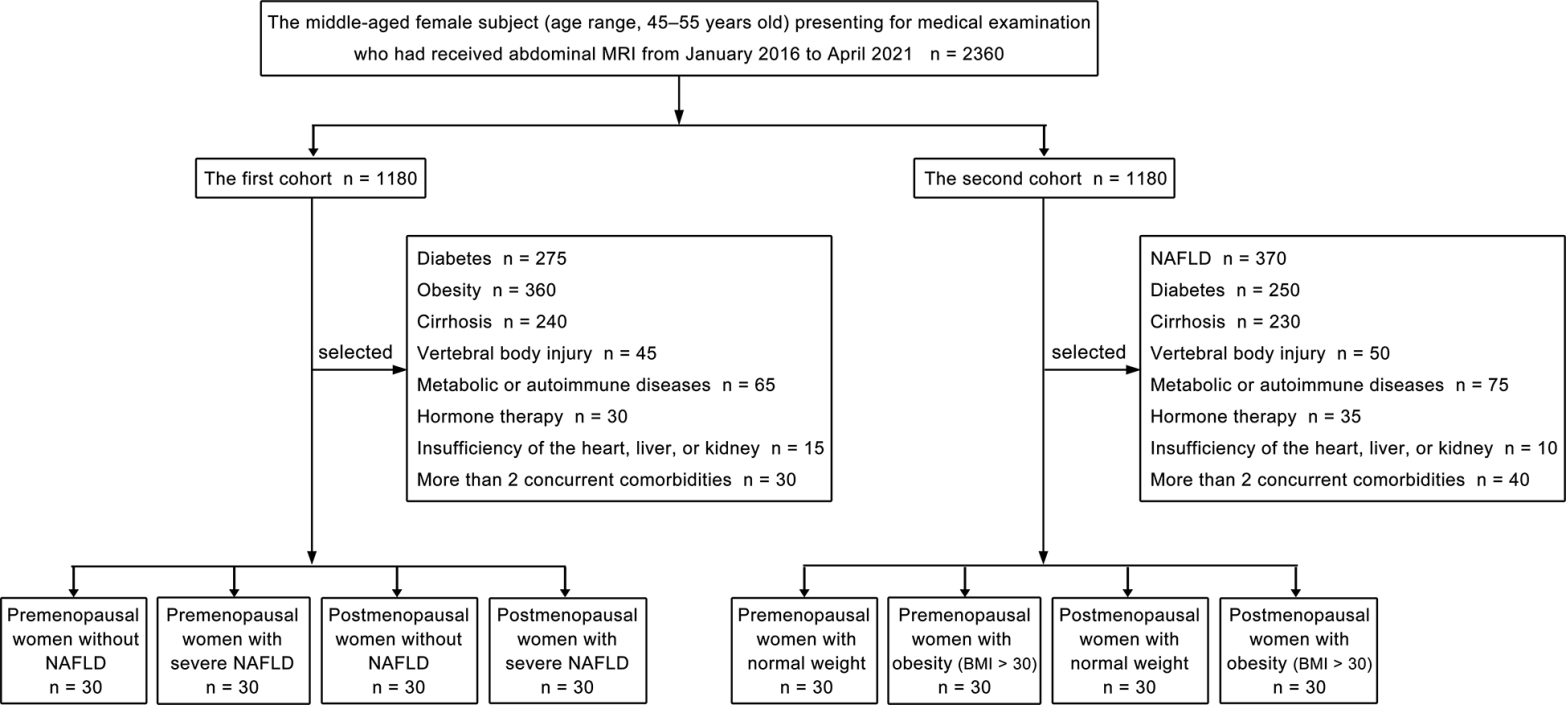
Flowchart of the patient selection process.

Our study consisted of two cohorts of patients, and each cohort included four groups. Propensity score matching was used to choose patients at a 1:1 ratio for the two cohorts’ patients, followed by subgrouping. The patient selection flowchart is shown in **Figure 1**. Women were categorized as postmenopausal if they had no menstrual cycle in the previous 12 months, whether owing to natural cessation or hysterectomy and/or oophorectomy (27). The first part of our study included the following four groups: premenopausal women without NAFLD (liver fat content is less than 5%) (28), premenopausal women with severe NAFLD (liver fat content is greater than 28%) (28), postmenopausal women without NAFLD, and postmenopausal women with severe NAFLD. Thirty patients were included in each group, and we quantitatively measured the fat deposition in the lumbar vertebra and liver of patients from each group. Patients with diabetes; obesity; cirrhosis; vertebral body injury; metabolic or autoimmune diseases; hormone therapy; insufficiency of the heart, liver, or kidney; or more than 2 concurrent comorbidities were excluded from the first cohort. The second cohort of patients in our study included the following four groups: premenopausal women with normal weight (body mass index, [BMI] 18- < 25 kg/m^2^) (29), premenopausal women with obesity (BMI > 30 kg/m^2^) (29), postmenopausal women with normal weight, and postmenopausal women with obesity (BMI > 30 kg/m^2^). Thirty patients were included in each group, and the fat deposition in the lumbar vertebra and subcutaneous fat were measured quantitatively. Patients with NAFLD; diabetes; cirrhosis; vertebral body injury; metabolic or autoimmune diseases; hormone therapy; insufficiency of the heart, liver, or kidney; or more than 2 concurrent comorbidities were excluded from the second cohort.

Of the 1180 consecutive patients in the first cohort, 1060 were excluded due to diabetes (n = 275), obesity (n = 360), cirrhosis (n = 240), vertebral body injury (n = 45), metabolic or autoimmune diseases (n = 65), hormone therapy (n = 30), insufficiency of the heart, liver or kidney (n = 15), or the presence of more than 2 concurrent comorbidities (n = 30). Accordingly, 120 women (mean age ± standard deviation, 49.50 ± 4.95 years old; range, 45 - 55 years old) were finally included in the first cohort. Of the 1180 consecutive patients in the second cohort, 1060 were excluded due to NAFLD (n = 370), diabetes (n = 250), cirrhosis (n = 230), vertebral body injury (n = 50), metabolic or autoimmune diseases (n = 75), hormone therapy (n = 35), insufficiency of the heart, liver or kidney (n = 10), or the presence of more than 2 concurrent comorbidities (n = 40). Accordingly, 120 women (mean age ± standard deviation, 49.00 ± 2.83 years old; range, 45 - 55 years old) were finally included in the second cohort. Significant differences in the mean age were not observed among the four groups (30 patients in each group) in the first or second cohorts of our study (**Tables 1**, **2**).

**Table 1:** Clinicopathologic characteristics of 120 patients in the first cohort.

Characteristics	Severe NAFLD-pre	Without NAFLD-pre	Severe NAFLD-post	Without NAFLD-post	P values			
					Severe NAFLD-pre vs. Without NAFLD-pre	Severe NAFLD-post vs. Without NAFLD-post	Severe NAFLD-pre vs. Severe NAFLD-post	Without NAFLD-pre vs. Without NAFLD-post
No. of patients	30	30	30	30	–	–	–	–
Mean Age (years old)	49.37	49.30	49.13	49.77	0.93	0.37	0.75	0.53
Mean body weight(kg)	52.07	51.35	52.10	51.20	0.48	0.35	0.97	0.88
Mean height(m)	1.61	1.58	1.62	1.59	0.76	0.71	0.79	0.72
Mean BMI(kg/m^2^)	23.54	22.67	21.85	22.68	0.39	0.45	0.36	0.41

BMI, body mass index; NAFLD, non-alcoholic fatty liver disease; Severe NAFLD-pre, Premenopausal with severe NAFLD; Without NAFLD-pre, Premenopausal without NAFLD; Severe NAFLD-post, Postmenopausal with severe NAFLD; Without NAFLD-post, Postmenopausal without NAFLD.

**Table 2:** Clinicopathologic characteristics of 120 patients in the second cohort.

Characteristics	Obese-pre	Normal weight-pre	Obese-post	Normal weight-post	P values			
					Obese-pre vs. Normal weight-pre	Obese-post vs. Normal weight-post	Obese-pre vs. Obese-post	Normal weight-pre vs. Normal weight-post
No. of patients	30	30	30	30	–	–	–	–
Mean Age (years old)	49.70	49.10	49.07	49.90	0.41	0.25	0.38	0.27
Mean body weight(kg)	82.27	51.52	82.40	51.37	< 0.001	< 0.001	0.72	0.88
Mean height(m)	1.62	1.59	1.61	1.58	0.77	0.72	0.78	0.71
Mean BMI(kg/m^2^)	33.86	23.24	34.15	22.95	< 0.001	< 0.001	0.41	0.47

BMI, body mass index; Obese-pre, Premenopausal with obese; Normal weight-pre, Premenopausal with normal weight; Obese-post, Postmenopausal with obese; Normal weight-post, Postmenopausal with normal weight.

### Magnetic resonance imaging

This study was performed using a 3.0 T MRI scanner (Discovery 750 and Signa Architect, GE Healthcare, Milwaukee, Wisconsin, USA) equipped with a 32-channel, phased-array torso coil by two radiologists. Before scanning, patients were trained to hold their breath for > 20 s at the end of expiration. Prior to IDEAL, the routine sequences, liver acquisition with volume acceleration and fast spin–echo T2-weighted images (T2WI) with fat saturation of the abdomen were recorded. The detailed acquisition parameters are shown in Supplementary Material and listed in **Supplementary Table 1**.

The IDEAL-IQ sequence was used on the abdomen in the axis plane. The following six groups of images were obtained once the IDEAL sequence was scanned: in-phase image, out-of-phase image, pure water image, pure fat image, fat fraction (FF) map, and R2* relaxation rate image. The adipose contents of the fat infiltration regions were directly measured using the FF map, and the measurements represent the percentage of fat content in the local bone mass, SAT, and liver.

### Image analysis

The IDEAL datasets were transferred to the workstation and processed using Functool 6.3.1 software (GE Healthcare, Milwaukee, Wisconsin, USA). Two radiologists independently evaluated the MRI studies. For the quantitative analysis of the FF values of the liver (FF_liver_), the radiologists independently placed three circulars (20 mm diameter) regions of interest (ROIs) in the liver on a plane passing through the main portal vein division: (1) in the right lobe of the liver (segment 6), (2) in segment 4 of the liver and (3) in the left lobe of the liver (segment 2/3). All ROIs were placed in the liver, avoiding major vessels, ligaments, and bile ducts, ensuring that each ROI was surrounded by liver parenchyma. Subcutaneous fat was defined as adipose tissue measured from the abdominal muscle wall to the skin. For the quantitative analysis of the FF values of SAT (FF_SAT_), box-shaped ROIs were placed on the left, middle, and right levels of the anterior abdominal subcutaneous fat in the upper abdomen. The ROIs were approximately 20 mm^3^. For the quantitation of FF values of the lumbar vertebra (FF_lumbar_), box-shaped ROIs were placed on each lumbar vertebra at the upper, middle, and lower levels (L1–L4) on the axial FF map, which avoids tumor diseases, bone island, Schmorl’s node, etc. in the vertebral body. The ROIs were approximately 20 mm^3^. Each area was measured 3 times, and the mean value was calculated. The liver fat content is less than 5% in the normal population and greater than 28% in patients with severe NAFLD (29).

### Statistical analysis

All the data are presented as the means ± standard deviations, and a *P value* of less than 0.05 was considered significant. Differences in FF_liver_, FF_SAT_, and FF_lumbar_ were analyzed using t tests with Bonferroni’s correction or the Mann–Whitney U test. The correlation coefficients were calculated by Spearman rank between the FF_liver_, FF_SAT_, and FF_lumbar_. The interobserver consistency of the FF values (independently determined by two radiologists) was evaluated using the Bland–Altman analysis and the intraclass correlation coefficient (ICC). One author analyzed all data using GraphPad Prism 9 software (GraphPad Software Inc., San Diego, CA, USA).

## Results

**Figure 2:**
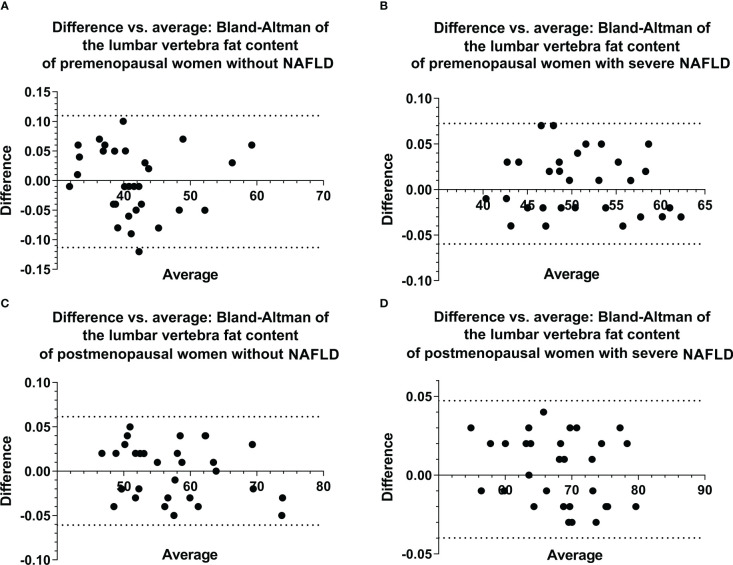
Bland–Altman plots of ROI-based FF values for the lumbar vertebra were obtained from two observations of the first cohort **(A–D)**, showing excellent correlations, a negligible bias, and 95% limits of agreement of approximately ± 0.113 or less. ROI, region of interest, FF, fat fraction.

The images of all patients were clear and usable. The interobserver consistency of the FF_lumbar_ in the first cohort of patients was analyzed by constructing a Bland–Altman plot (**Figure 2**), and the 95% limits of agreement were -0.11 to 0.11, -0.06 to 0.06, -0.06 to 0.07, and -0.04 to 0.05, respectively. For the quantitative measurement of FF_lumbar_ in premenopausal without NAFLD group and premenopausal with severe NAFLD group, interobserver agreement was high (ICC = 0.91, 0.93; 95% confidence interval (CI) 0.90–0.93, 0.91–0.95), and intraobserver agreement was also high (ICC = 0.92, 0.92; 95% CI 0.91–0.94, 0.90–0.94). The Bland–Altman analysis and ICC showed that the two radiologists had good consistency in the measured values.

**Figure 3:**
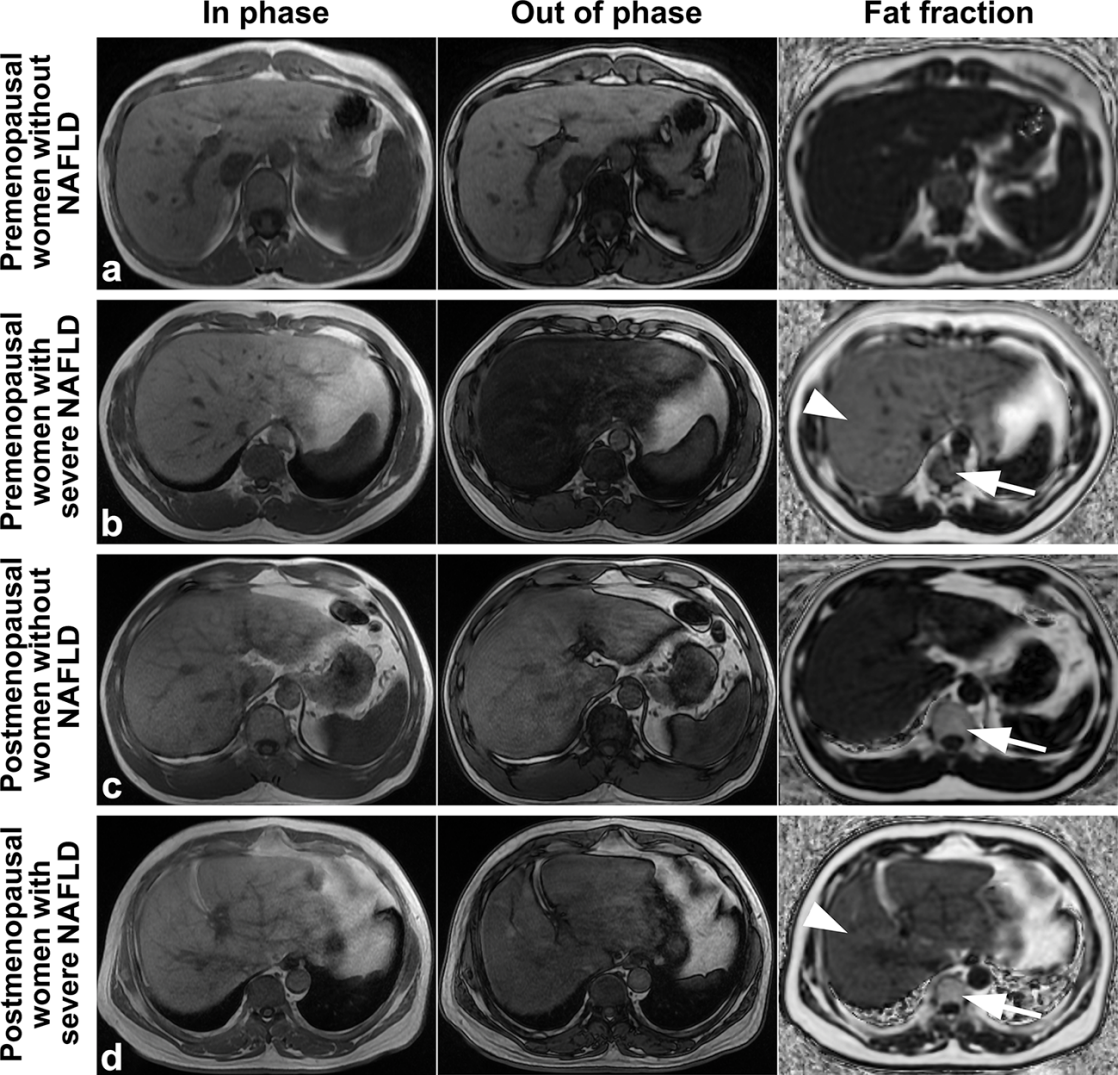
Images of premenopausal and postmenopausal women without NAFLD or with severe NAFLD **(A)** a 47 years old premenopausal woman without NAFLD; **(B)** a 45 years old premenopausal woman with severe NAFLD; **(C)** a 55 years old postmenopausal woman without NAFLD; and **(D)** a 57 years old postmenopausal woman with severe NAFLD). The fat deposition was visible by the decrease in the signal in the axial conventional T1-in phase and out-of-phase images of the mDIXON-Quant sequence and was measured in the fraction map of the IDEAL-IQ sequence. The example showed that fat deposition in a lumbar vertebra (arrows) increased after menopause in patients without NAFLD or with severe NAFLD (arrowheads). IDEAL-IQ, iterative decomposition of water and fat with echo asymmetry and least-squares estimation.

**Figure 4:**
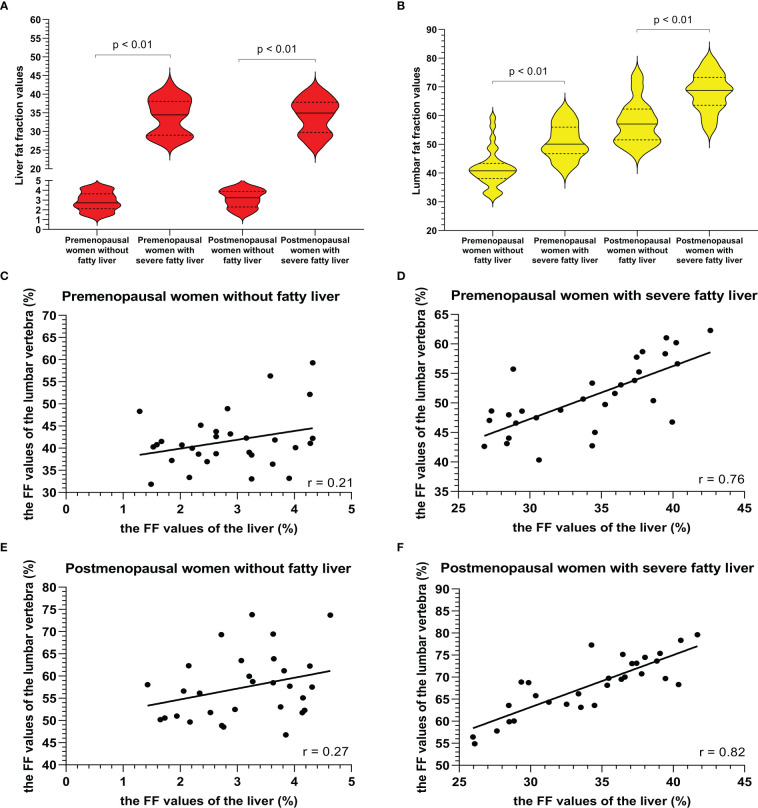
The violin plot analyzing the fat fraction values for the liver fat content **(A)** and lumbar fat content **(B)** in four groups (premenopausal women without NAFLD, premenopausal women with severe NAFLD, postmenopausal women without NAFLD, and postmenopausal women with severe NAFLD; 30 subjects in each group) confirmed that the lumbar fat content of patients with severe NAFLD more than that without NAFLD before menopause (*p* < 0.01) and after menopause (*p* < 0.01). The correlation plots for the correlations between FF_lumbar_ and the FF_liver_ **(C–F)** The correlation between FF_lumbar_ and the FF_liver_ in premenopausal **(C)** or postmenopausal **(E)** women without NAFLD was no different. A high correlation was detected between FF_lumbar_ and the FF_liver_ in women with severe NAFLD (premenopausal women: r = 0.76, *p* < 0.01; postmenopausal women: r = 0.82, *p* < 0.01) **(D, F)**.

Examples of fat deposition in patients without NAFLD and severe NAFLD before or after menopause in the first part are shown in **Figure 3**. The mean FF_lumbar_ and the FF_liver_ were measured separately in the four groups and are summarized in **Table 3**. In premenopausal women and postmenopausal women, the FF_lumbar_ were higher in patients with severe NAFLD than in patients without NAFLD (premenopausal women: 50.95 ± 6.03 vs. 41.58 ± 6.37, *p* < 0.001, t = 5.85; postmenopausal women: 68.11 ± 6.49 vs. 57.48 ± 7.38, *p* < 0.001, t = 5.92). Additionally, in patients with severe NAFLD or without NAFLD, the FF_lumbar_ were higher in postmenopausal women than in premenopausal women (severe NAFLD: 68.11 ± 6.49 vs. 50.94 ± 6.03, *p* < 0.001, t = 10.62; without NAFLD: 57.48 ± 7.38 vs. 41.58 ± 6.37, *p* < 0.001, t = 8.93). Significant differences in the FF_lumbar_ were observed between patients without NAFLD and with severe NAFLD in both premenopausal and postmenopausal women (**Figures 4A, B**). A high correlation was detected between FF_lumbar_ and the FF_liver_ in women with severe NAFLD (premenopausal women: r = 0.76, *p* < 0.01; postmenopausal women: r = 0.82, *p* < 0.01) (**Table 4**; **Figures 4C–F**). The correlation between FF_lumbar_ and the FF_liver_ was better in postmenopausal women with severe NAFLD than in premenopausal women with severe NAFLD.

**Table 3:** The fat fraction values of the lumbar vertebra and liver in the first cohort.

Characteristics	Severe NAFLD-pre	Without NAFLD-pre	Severe NAFLD-post	Without NAFLD-post	P values			
					Severe NAFLD-pre vs. Without NAFLD-pre	Severe NAFLD-post vs. Without NAFLD-post	Severe NAFLD-pre vs. Severe NAFLD-post	Without NAFLD-pre vs. Without NAFLD-post
FF_lumbar_ (mean ± SD)	50.95 ± 6.03	41.58 ± 6.37	68.11 ± 6.49	57.48 ± 7.38	= 0.001	= 0.001	= 0.001	= 0.001
FF_liver_ (mean ± SD)	34.10 ± 4.79	2.85 ± 0.94	34.17 ± 4.60	3.13 ± 0.91	= 0.001	= 0.001	0.95	0.24

SD, standard deviation; FF_liver_, fat fraction values of the liver; FF_lumbar_, fat fraction values of lumbar; NAFLD, non-alcoholic fatty liver disease; Severe NAFLD-pre, Premenopausal with severe NAFLD; Without NAFLD-pre, Premenopausal without NAFLD; Severe NAFLD-post, Postmenopausal with severe NAFLD; Without NAFLD-post, Postmenopausal without NAFLD.

**Figure 5:**
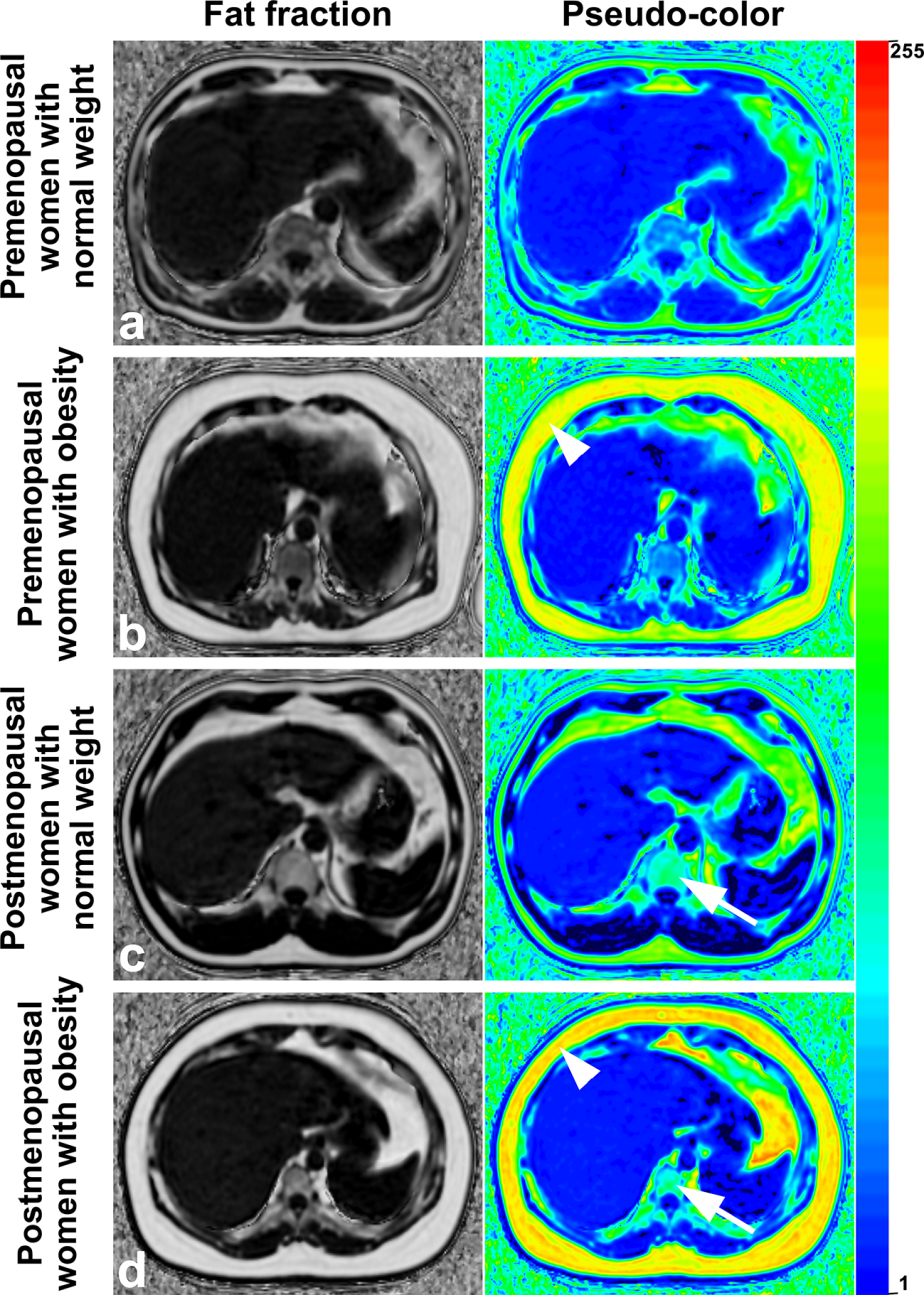
Images of premenopausal and postmenopausal women with obesity or normal weight patients **(A)** a 46 years old premenopausal woman with normal weight; **(B)** a 44 years old premenopausal woman with obesity; **(C)** a 53 years old postmenopausal woman with normal weight; and **(D)** a 56 years old postmenopausal woman with obesity). Fat deposition in subcutaneous sites (arrowheads) and lumbar vertebrae (arrows) was visible and was measured in the fraction map and pseudocolor images of the IDEAL-IQ sequence. Examples showed that fat deposition in the lumbar vertebra increased after menopause in both obese patients and normal-weight patients. IDEAL-IQ, iterative decomposition of water and fat with echo asymmetry and least-squares estimation.

**Figure 6:**
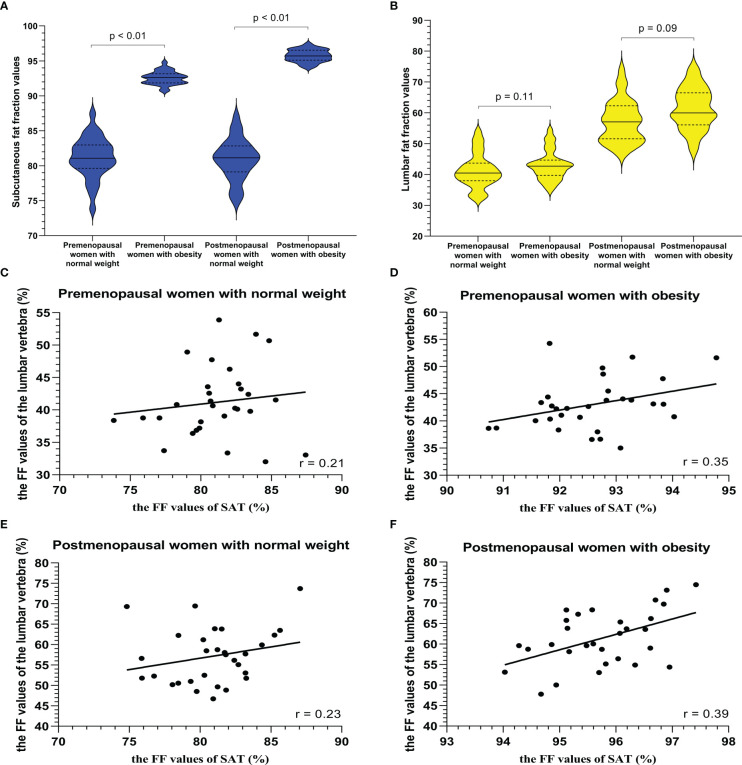
The violin plot analyzing fat fraction values for a subcutaneous site **(A)** and lumbar vertebra **(B)** in four groups (premenopausal women with normal weight, premenopausal women with obesity (BMI > 30 kg/m2), postmenopausal women with normal weight, and postmenopausal women with obesity (BMI > 30 kg/m2); 30 subjects in each group) confirmed that there is no statistical difference between the lumbar fat content of obese patients and that of normal-weight patients before menopause (*p* = 0.11) and after menopause (*p* = 0.09), and the subcutaneous fat content in the obese patients more than that in normal weight subjects (*p* < 0.01). BMI = body mass index. The correlation plots for the correlations between FF_lumbar_ and the FF_SAT_ **(C–F)** The correlation between FF_lumbar_ and the FF_SAT_ in premenopausal or postmenopausal women without or with obesity was no different **(C–F)**.

Examples of fat deposition in obese patients and normal-weight patients in the second cohort before or after menopause are shown in **Figure 5**. The mean FF_lumbar_ and the FF_SAT_ were measured separately in the four groups and are summarized in **Table 5**. Both in premenopausal women and postmenopausal women, no significant difference in the FF_lumbar_ was observed obese patients and in normal-weight patients (premenopausal women: 44.15 ± 4.73 vs. 41.17 ± 5.49, *p* = 0.113, t = 5.18; postmenopausal women: 61.39 ± 6.75 vs. 57.15 ± 6.83, *p* = 0.092, t = 6.42). Postmenopausal women had higher FF_lumbar_ than premenopausal women, regardless of the presence of obesity (obese: 61.39 ± 6.75 vs. 42.98 ± 4.73, *p* < 0.001, t = 12.23; normal weight: 57.15 ± 6.83 vs. 41.17 ± 5.49, *p* < 0.001, t = 9.99). Significant differences in the FF values of lumbar vertebra were observed between premenopausal women and postmenopausal women, but not between patients with obesity or without obesity (**Figures 6A, B**). No statistically significant correlation was found between the FF_lumbar_ and the FF_SAT_ in this cohort (**Table 4**; **Figures 6C-F**).

**Table 4:** The correlation coefficients between the FF_liver_, FF_SAT_, and FF_lumbar_.

	Group	r	95% confidence interval	p value
**FF_liver_ vs. FF_lumbar_**	Premenopausal women without NAFLD	0.21	-0.10 to 0.53	0.13
	Premenopausal women with severe NAFLD	0.76	0.51 to 0.89	< 0.01
	Postmenopausal women without NAFLD	0.27	-0.11 to 0.58	0.15
	Postmenopausal women with severe NAFLD	0.82	0.65 to 0.91	< 0.01
**FF_SAT_ vs. FF_lumbar_**	Premenopausal women with normal weight	0.21	-0.12 to 0.51	0.28
	Premenopausal women with obesity	0.35	0.07 to 0.63	0.19
	Postmenopausal women with normal weight	0.23	-0.15 to 0.56	0.22
	Postmenopausal women with obesity	0.39	0.12 to 0.69	0.17

FF_liver_, fat fraction values of the liver; FF_SAT_, fat fraction values of subcutaneous adipose tissue; FF_lumbar_, fat fraction values of lumbar; NAFLD, non-alcoholic fatty liver disease.

**Table 5:** The fat fraction values of lumbar vertebra and subcutaneous fat in the second cohort.

Characteristics	Obese-pre	Normal weight-pre	Obese-post	Normal weight-post	P values			
					Obese-pre vs. Normal weight-pre	Obese-post vs. Normal weight-post	Obese-pre vs. Obese-post	Normal weight-pre vs. Normal weight-post
FF_lumbar_ (mean ± SD)	44.15 ± 4.73	41.17 ± 5.49	61.39 ± 6.75	57.15 ± 6.83	0.11	0.09	= 0.001	= 0.001
FF_SAT_ (mean ± SD)	92.57 ± 0.92	81.15 ± 2.90	95.74 ± 0.87	80.88 ± 2.92	= 0.001	= 0.001	0.41	0.73

SD, standard deviation; SAT, subcutaneous adipose tissue; FF_SAT_, fat fraction values of subcutaneous adipose tissue; FF_lumbar_, fat fraction values of lumbar; Obese-pre, Premenopausal with obese; Normal weight-pre, Premenopausal with normal weight; Obese-post, Postmenopausal with obese; Normal weight-post, Postmenopausal with normal weight.

## Discussion

Our study focused on the association between abdominal adipose tissue and vertebral marrow fat in middle-aged women using the IDEAL-IQ methodology. Our analyses indicated that the vertebral marrow fat content was increased in postmenopausal women and that liver fat deposition potentially aggravated this situation. However, no statistically significant correlation was observed between vertebral marrow fat and subcutaneous fat deposition.

Traditionally, adipose tissue was postulated to exert a protective effect on bone. Researchers have speculated that this protective effect may be due to the stimulation of bone formation by the high mechanical load associated with overweight and obesity (11). However, a recent study showed that different local fat compartments are responsible for different metabolic effects and different effects on bone (30, 31). Wang et al. indicated that SAT has no relation to bone mineral density (BMD) in Chinese women < 55 years old (32). In contrast, Melissa et al. revealed that VAT and SAT had inverse associations with BMD in obese adolescent girls, with SAT exhibiting positive associations and VAT showing negative associations (33). In our study, SAT had no correction with vertebral marrow fat in postmenopausal women. Our results are consistent with a study of older Chinese women (32). However, the results are different from studies of obese adolescent girls (30, 33). This difference is probably because the population age in our study was much older than that in the studies of young girls, and the estrogen level of the population in our study was much lower than that in young girls.

NAFLD is strongly associated with abdominal obesity and metabolic disturbances (13). Preliminary data also suggest that NAFLD may be associated with other common and chronic debilitating conditions, particularly low BMD (14, 34). Our study found that liver fat deposition potentially aggravated vertebral marrow fat content in postmenopausal women. Adipose-modulated biochemical signals may explain some associations between fat mass and bone metabolism. Adipose tissue secretes various inflammatory cytokines and hormones, such as tumor necrosis factor-α and interleukin-6. These inflammatory cytokines promote osteoclast differentiation and activation and inhibit osteoclast apoptosis (34, 35). NAFLD may participate in bone metabolism *via* the systemic release of multiple proinflammatory, procoagulant, pro-oxidant and profibrogenic mediators and/or *via* the direct effect on hepatic and systemic insulin resistance (35, 36). The previousstudy also reported a positive correlation between hepatic fat content and bone marrow fat content in children with known or suspected NAFLD (37). However, the correlation in our study was much higher than that in this previous study. This difference is probably due to the subjects enrolled were not the same. In our study, subjects were over 45 years old which was much older than that in the previous. Besides, the patients with severe NAFLD were enrolled in our study. Estrogen level and varying degrees of NAFLD severity may be the reason for the above results.

Osteoporosis is characterized by a low BMD and progressive deterioration of the bone microarchitecture. The fat content in bone marrow is negatively correlated with BMD because the lost bone mass in the vertebral space is infilled with fatty bone marrow (38, 39). Therefore, the increase in vertebral marrow fat may reflect the progression of osteoporosis. Our findings are consistent with previous studies revealing that the menopause transition is associated with increased central adiposity (6, 36, 40) and confirmed a higher incidence of osteoporosis in postmenopausal women than in premenopausal women.

Our study has several limitations. First, our study was a retrospective cross-sectional study. As a cross-sectional study, we are unable to establish a causal relationship between liver fat and vertebral marrow fat. A further longitudinal prospective study with a large sample size is warranted to validate the current findings. Second, we were unable to control for the potential factors affecting bone loss and vertebral marrow fat deposition, such as dietary calcium intake or vitamin D supplementation. Third, this study did not focus on the thickness or volume of subcutaneous fat but on the percentage of fat in subcutaneous fat (mainly composed of fat and water). Finally, our study population included only middle-aged women, and our findings cannot be extrapolated to young women or the male skeleton. This approach limits the generalizability of the results but should not affect the internal validity.

## Conclusions

This study describes a precise and noninvasive IDEAL-IQ technology to measure the fat content of vertebral marrow, SAT, and liver in pre- or postmenopausal women. FF_lumbar_ was significantly higher in postmenopausal women than in premenopausal women. FF_lumbar_ was higher in patients with severe NAFLD than in patients without NAFLD, but FF_lumbar_ was not significantly different between obese patients with subcutaneous fat deposition and normal-weight patients, indicating that fat deposition in the vertebral marrow was significantly associated with liver fat deposition in postmenopausal women with severe NAFLD. Furthermore, our findings support the hypothesis that liver fat deposition is relative to vertebral fat deposition (which may cause osteoporosis) in postmenopausal women.

## Data availability statement

The original contributions presented in the study are included in the article/**Supplementary Material**. Further inquiries can be directed to the corresponding authors.

## Ethics statement

The studies involving human participants were reviewed and approved by the Institutional Research Ethics Committee of the Third Affiliated Hospital of Sun Yat-Sen University (02-005-01). The patients/participants provided their written informed consent to participate in this study.

## Author contributions

C-SZ, R-MG: Data collection, Data analysis, Manuscript writing. H-QW, W-SL, X-WL, F-YZ, and XZ: Data collection. H-JH, Q-LL, L-SS, and R-MG: Data analysis, Manuscript editing. R-MG: Project development, Data analysis, Manuscript editing. All authors contributed to the article and approved the submitted version.
