# Supplementary material for: Quantification of lumbar vertebral fat deposition: Correlation with menopausal status, non-alcoholic fatty liver disease and subcutaneous adipose tissue
Source: Front Endocrinol (Lausanne). 2023 Jan 12;13:1099919. doi: 10.3389/fendo.2022.1099919 (PMC9878446; doi:10.3389/fendo.2022.1099919)
Supplement: Supplementary file 1 [file Table_1.docx]

**Supplementary Table 1 MRI acquisition parameters**

|  | T1WI | T2WI | IDEAL-IQ |
| --- | --- | --- | --- |
| TR (ms) | **4.6** | **6666** | **6.4** |
| TE (ms) | **2.3** | **70** | **0.8** |
| FOV (cm) | **40×40** | **40×40** | **40×40** |
| Slice Thickness (mm) | **5** | **5** | **5** |
| Interslice Gap (mm) | **-** | **1** | **-** |
| Acquisition Matrix | **384×384** | **512×512** | **128×128** |
| NEX | **1** | **2** | **0.5** |
| BandWidth (kHz) | **142.86** | **83.33** | **125** |
| Flip Angle | **15** | **111** | **3** |
| Acceleration | **2** | **2** | **2** |
| Acquisition Time | **15s** | **3min15s** | **18s** |

TR: repetition time; TE: echo time; FOV: field of view; NEX: number of excitations.
